# *In Vivo* Detection of Macrophage Recruitment in Hind-Limb Ischemia Using a Targeted Near-Infrared Fluorophore

**DOI:** 10.1371/journal.pone.0103721

**Published:** 2014-07-29

**Authors:** Jung Sun Yoo, Raj Kumar Das, Zhi Yen Jow, Young-Tae Chang

**Affiliations:** 1 Smart Humanity Convergence Center, Department of Transdisciplinary Studies, Graduate School of Convergence Science and Technology, Seoul National University, Suwon, Korea; 2 Department of Chemistry, National University of Singapore, Singapore, Singapore; 3 Laboratory of Bioimaging Probe Development, Singapore Bioimaging Consortium, Agency for Science, Technology and Research, Singapore, Singapore; Singapore Immunology Network, Agency for Science, Technology and Research (A*STAR), Singapore

## Abstract

Macrophages are an essential component of the immune system and have protective and pathogenic functions in various diseases. Imaging of macrophages *in vivo* could furnish new tools to advance evaluation of disease and therapies. Critical limb ischemia is a disease in which macrophages have considerable pathogenic roles, and are potential targets for cell-based immunotherapy. We sought to develop a new near-infrared fluorescence (NIRF) imaging probe to target macrophages specifically *in* *vivo* in various pathological states, including hind-limb ischemia. We rapidly screened the photostable cyanine-based NIRF library against different blood cell lines. The identified monocyte/macrophage-selective hit was tested *in* *vitro* in live-cell labeling assay. Non-invasive NIRF imaging was performed with murine models of paw inflammation by lipopolysaccharide challenge and hind-limb ischemia with femoral artery ligation. *in* *vivo* macrophage targeting was further evaluated using intravital microscopy with Csf1r-EGFP transgenic mice and immunofluorescent staining with macrophage-specific markers. We discovered MF800, a Macrophage-specific near-infrared Fluorophore, which showed selective live-cell imaging performance in a panel of cell lines and primary human blood samples. MF800 outperforms the clinically-available NIRF contrast agent ICG for *in* *vivo* specificity in paw inflammation and hind-limb ischemia models. We observed a marked overlap of MF800-labeled cells and EGFP-expressing macrophages in intravital imaging of Csf1r-EGFP transgenic mice. In the histologic analysis, MF800-positive cells also expressed the macrophage markers CD68 and CD169. NIRF imaging showcased the potential of using MF800 to understand macrophage behavior *in* *vivo*, characterize macrophage-associated diseases, and may help in assessing therapeutic responses in the clinic.

## Introduction

Macrophages have a multitude of roles in biology, from development, homeostasis, and tissue repair to immunity. They also play several important roles in pathological processes and, thus, have emerged as therapeutic targets or prognostic indicators in many diseases [1], [2], [3]. Cardiovascular disease is one of the world’s leading causes of morbidity and mortality, and in particular, peripheral arterial occlusive diseases, such as critical limb ischemia, which affects one in five individuals over the age of 75 [4]. Occlusions of arteries, by atherosclerotic lesions or thrombi, cause insufficient blood supply and intractable pain, and often resulting in ulceration or gangrene, leading to amputation of the limb [5]. Interestingly, monocytes/macrophages are pivotal players in the remodeling process by becoming angiogenic cells after ischemic events and promoting collateral arterial development [6], [7]. Therefore, specific detection of macrophage content *in* *vivo* could lead to more accurate assessment of repair and treatment. In recent years, cell-based therapy using monocytes/macrophages has received particular attention as a means for sustainable production of various growth factors required for effective revascularization [8], [9], [10]. Methods are thereby needed to target macrophages with high sensitivity and selectivity under *in* *vivo* conditions.

Although several imaging techniques, such as those using magnetic resonance imaging (MRI) and positron emission tomography (PET), have been developed [11], near-infrared fluorescence (NIRF) imaging is evolving into a promising approach to monitor macrophages *in* *vivo* in real time. Light in the NIR spectrum (wavelength: 650–900 nm) improves tissue penetration of the signal and simultaneously reduces autofluorescence of the tissue [12], [13]. These attractive characteristics, together with the inherent advantages of optical imaging, such as high temporal and spatial resolution, no ionizing radiation, small and relatively cheap equipment, and convenient histological detection, have facilitated vibrant developments of *in* *vivo* NIRF imaging techniques [14], [15], [16]. NIRF nanoparticle-based [17], [18] or quantum dot-based [19] imaging is one strategy for identifying macrophages with *in* *vivo* NIRF imaging. Although macrophages appear to internalize nanoparticles effectively because of favorable size (10–50 nm) and dextran coating, a relatively long targeting time (more than 24 hours) and an apparently low selectivity have been major drawbacks of nanoparticle-based macrophage imaging [20]. Another strategy utilizes activatable probes to detect a range of proteases, such as matrix metalloproteinases and cathepsin B, produced by macrophages [3], [21]. Such probes minimize background and record clinically-relevant information for inflammatory diseases. However, enzyme activity does not always co-localize with macrophage location and distribution in single-cell level, which limits proper cellular targeting [22]. In addition, protease activity is not associated with the tissue-repair by macrophages.

Organic fluorochromes are affordable for clinical cell-labeling and have a favorable toxicity profile and blood half-life [11]. Recently, NIRF imaging of macrophages has been demonstrated in atherosclerosis models using indocyanine green (ICG), a Food and Drug Administration-approved NIR fluorochrome [23]. This study showed that ICG could target lipid-loaded macrophages in atheromas, owing to its lipophilic properties, suggesting that a vital dye-based approach is appropriate for *in* *vivo* macrophage visualization. A distinct drawback is that ICG has poor fluorescent properties, including significant instability [14]. As ICG binds to plasma lipoprotein and is internalized within macrophages, its applications are also restricted to lipid-rich inflamed regions. To address the limitations, we first synthesized cyanine-based NIRF dye libraries by solid-phase chemistry and succeeded to achieve superior photostability in comparison to ICG [24], [25]. Given this promising characteristics, we then hypothesized that a high-throughput screen with the developed libraries would lead to a highly pertinent NIRF imaging probe that targets macrophages selectively in a pathological environment.

Here, we describe the development of a Macrophage-specific near-infrared Fluorophore (MF800) that may be used for diagnosis and for monitoring response to therapy. MF800 is capable of selectively staining monocytes/macrophages ranging from cell lines to human peripheral blood-derived cells for live-cell microscopy. In combination with *in* *vivo* imaging and intravital microscopy, MF800-mediated NIRF directly targets macrophages in an LPS-induced local inflammation model, as well as a clinically-relevant hind-limb ischemia model, with superior specificity over ICG. We believe that utilizing MF800 for NIRF macrophage imaging could prove valuable in evaluating the behavior of macrophages *in* *vivo*, and clinical monitoring of disease progression, including ischemia.

## Materials and Methods

### Preparation of cells

MOLT-4 and U-937 cells were purchased from the American Type Culture Collection (ATCC) and were grown in RPMI 1640 medium with 10 % fetal bovine serum (FBS), 1× GlutaMAX, 1× non-essential amino acid, 100-U/ml penicillin, and 100-µg/ml streptomycin (Life Technologies). The non-adherent monocyte-like U-937 cells (2×10^5^/mL) were treated with 150 nM of phorbol-12-myristate-13-acetate (PMA, Sigma) for differentiation into U-937-derived macrophages (U-937-DM). After 24 hours, non-adherent cells were removed by a gentle washing with phosphate buffered saline (PBS). Fresh whole blood samples were obtained from consenting healthy human donors. The National University of Singapore Institutional Review Board (NUS-IRB Reference code 12–195) approved the studies, and subjects gave written, informed consent.

Mononuclear cells and granulocytes were isolated by using Ficoll Paque (GE Healthcare) density-gradient centrifugation. First, the anticoagulant-treated whole blood sample diluted with Hank’s balanced salt solution with 2 mM EDTA was carefully layered onto the Ficoll-Paque media solution. After centrifuge at 400 g for 40 min at 18°C, we draw off the upper layer containing plasma and platelets using a sterile pipette and then transferred the layer of mononuclesr cells to a sterile centrifuge tube, not disturbing the lower layer of granulocytes on the top of the erythrocytes. Finally, the layer containing granulocytes and a bit of erythrocytes were transferred to another sterile centrifuge tube. T lymphocytes and monocytes were separated from the freshly-isolated mononuclear cells by using negative magnetic-bead sorting (Human T Lymphocyte Enrichment Set-DM #557874, Human Monocyte Enrichment Set-DM #558454, BD Biosciences), both with more than 84 % purity measured by immuno-flow cytometry. Freshly-purified monocytes (1–1.5×10^6^/mL) were further differentiated into monocyte-derived macrophages using RPMI 1640 medium containing 10 % whole human serum, 1x GlutaMAX, 100-U/ml penicillin, 100-µg/ml streptomycin and 200-U/ml macrophage colony-stimulating factor (M-CSF, R&D Systems) for 5 days. Granulocytes were purified from the transferred mixture of granulocytes and erythrocytes using lysis buffer (154 mM NH_4_Cl, 88 mM KNCO_3_, 127 µM EDTA) to remove erythrocytes.

### Chemical libraries and screening

The cyanine- and tetraarylazadipyrromethene-based NIR fluorescence dye libraries (CyR, CyNA and aza-BODIPY) were synthesized as previously reported [24], [25], [26] by a diversity-oriented approach, and in total, 520 fluorescent compounds were screened against lymphocytes (MOLT-4) and monocytes/macrophages (U-937, U-937-DM) by high-throughput flow cytometry. U-937-DMs were dissociated and collected using 0.25 % trypsin-ethylenediaminetetraacetic acid (EDTA). Cells were plated onto non-coated 384-well plates at a density of 10000 cells/well in culture medium. A mild cell detachment solution (Accumax, Stem Technologies) was added for U-937-DMs to avoid cell attachment during analysis. Cells were exposed to the fluorescent compounds at a final concentration of 0.5 µM or 1 µM in a volume of 100 µl per well containing 0.1% dimethyl sulfoxide (DMSO, v/v) for 1 h at 37°C, and were then analyzed for NIR fluorescence expression in the APC-Cy7 channel using an automated high-throughput flow cytometer (LSRFortessa, BD Biosciences). The primary hit compounds were selected, using the FACSDiva software (BD Biosciences), based on the criteria that the mean values of the fluorescence intensities for monocytes/macrophages be greater than five standard deviations above the mean intensity of lymphocytes.

### MF800 synthesis

A detailed description of the synthesis of the MF800 can be found in the Supplementary Methods in File S1. The synthesized MF800 was characterized by using liquid chromatography-mass spectrometry (LC-MS), and both ^1^H and ^13^C nuclear magnetic resonance (NMR) (Figs. S2–S4 in File S1). The quantum yield was calculated using Cardiogreen as a standard (Supplementary Methods in File S1).

### Cellular imaging

Cells were incubated in the culture medium at 37°C and 5 % CO_2_ for 1 hour in the presence of 2-µM MF800 for cell lines (MOLT-4, U-937, U-937-DM) and 6-µM MF800 for human peripheral blood-derived cells. Hoechst 33342 was also added for nuclear counterstaining. Following incubation, fluorescence images were obtained with an Eclipse Ti-E inverted microscope (Nikon) equipped with a 750 nm NIR light-emitting diode (LED) light source (pE-100, CoolLED), a 750/20 nm band-pass excitation filter, a 785 nm long-pass dichroic mirror, and a 800/20 nm band-pass emission filter. All filters were purchased from Chroma Technology Corp. All NIR fluorescence images were obtained with identical measurement parameters and contrasts. The mean intensity was calculated from three separate experiments after normalization to the peak signal using Matlab software (MathWorks).

### *In vivo* mouse imaging

All procedures involving mice were approved by the Institutional Animal Care and Use Committee of the Biological Resource Center (BRC, Biomedical Sciences Institute, A*STAR) and were conducted in accordance with the BRC regulations concerning the care and use of experimental animals. C57BL/6-*Tg(Csf1r-EGFP-NGFR/FKBP1A/TNFRSF6)2Bck/J* mice which have the whole mononuclear phagocyte lineage labeled [27], were purchased from The Jackson Laboratory, and C57BL/6 mice were obtained from the BRC. For local inflammation models, 5 mg per kg of body weight of lipopolysaccharide (Sigma) in saline was injected into the right paw subcutaneously 24 hours before the imaging experiments. Models of hind-limb ischemia were generated by surgical ligation of the right femoral artery following a previously-reported protocol [28]. Three days after ligation, *in* *vivo* imaging experiments were performed.

Mice were anesthetized by intraperitoneal injection of ketamine (150 mg/kg) and xylazine (10 mg/kg). MF800 (100 µM in saline containing 1% of poly(ethylene glycol)4600(PEG) and 0.1 % of tween20, 10 µL/g) was injected intravenously (i.v.) in the LPS-induced local inflammation (n = 6) and hind-limb ischemia (n = 4) models 4 hours before imaging experiments. For controls, the same dose of ICG or the same amount of saline containing 1 % of PEG and 0.1 % of tween20 were injected instead. We also used saline-paw injected and sham-operated mice with MF800 injection. For sham-operated models, skin incision and suturing in the right leg were performed without ligation of the femoral artery.

The *in* *vivo* NIRF images were taken on an IVIS spectrum imaging system (Perkin Elmer, excitation: 745 ± 15 nm; emission: 800 ±10 nm). Intravital cell imaging was further performed with the hind-limb ischemia models after minimal skin incision. A stereofluorescence microscope system (M205FA, Leica) equipped with two charge-coupled device (CCD) cameras (Photometrics Evolve EMCCD, Leica DFC495 Color CCD) was used after modification for NIR and EGFP detection (NIR excitation: 740 ± 17.5 nm; NIR emission: 780 nm long pass filter; EGFP excitation: 480 ± 10 nm; EGFP emission: 510 ± 10 nm). Anesthesia was maintained with 1.5 % isoflurane/98.5 % O_2_ at 5 L/min, and the body temperature of the mice was kept constant at 37°C during all imaging procedures. Fluorescence reflectance imaging data obtained using the IVIS spectrum system were analyzed with Matlab software (MathWorks). The TBR was determined as the ratio of the mean intensity of the identified inflamed or ischemic region divided by that of the non-inflammation- or ischemia-induced counterpart. The regions of interest (ROIs) were manually chosen over the inflammation- or the ischemia- positive regions, as well as over the regions with inflammation- or ischemia-negative signals.

### Flow cytometry

Single-cell suspensions of hind-limb muscles containing the femoral arteries, and paw tissues were generated by careful mincing tissues and subsequent digestion at 37°C for 1 hour in DMEM (Life Technologies) in the presence of 250 µg/ml liberase CI (Roche) plus 50 µg/ml DNase I (Roche). After filtration through a 40 µm nylon cell strainer (BD Biosciences) and centrifugation for 5 min at 400 g, the cells were washed and treated with ammonium-chloride-based erythrocyte lysis buffer. All flow cytometric measurements were performed in the buffer containing PBS with 2 mM EDTA and 2 % FBS (Life Technologies) on a Becton Dickinson LSRFortessa flow cytometer and were analyzed by FlowJo software (Treestar Inc.). Csf1r-EGFP positive and MF800 positive signals have been detected through FITC/Alexa Fluor488 (Excitation: 488 nm laser with 50 mW power; Emission: 530 ± 15 nm filter) and APC-Cy7/APC-H7 (Excitation: 640 nm laser with 40 mW power; Emission: 780 ± 30 nm filter) channels, respectively.

### Immunofluorescence

After *in* *vivo* fluorescence imaging, C57BL/6 mice were euthanized and the limb muscles containing femoral arteries and paw tissues were dissected. The tissues were immersed in tissue freezing medium (TFM, Triangle Biomedical Sciences), frozen, and sectioned in 8 µm segments. The tissue sections were rinsed with PBS and fixed with 4 % paraformaldehyde for 20 min at room temperature (RT). After an additional series of washes with PBS, the tissue sections were cleared with 3 % sodium deoxycholate solution for 2 hours at RT, blocked with 20 % normal goat serum in 1 % BSA-PBS for 2 hours at 37°C, incubated with rat anti-mouse CD169 (1:200, Serotec) or rat anti-mouse CD68 (Abcam) at 4°C overnight (> 17 hours) diluted in 1 % BSA-PBS, rinsed three times with PBS, and incubated with Alexa Fluor 594 goat anti-rat IgG secondary antibody at RT for 2 hours. Then, the slides were washed with PBS several times, counterstained with Hoechst 33342 and mounted with ProLong Gold antifade reagent (Life Technologies). The images were captured with an Eclipse Ti-E inverted microscope (Nikon) modified for NIR fluorescence detection.

## Results

### Differential screening to identify a macrophage-specific NIRF probe

A total of 520 NIRF dyes, including photostable cyanine fluorophores [24], [25], were prepared from in-house small-molecule libraries from diversity-oriented synthesis. Using a high-throughput flow cytometer, we rapidly measured the fluorescence intensity of each incubated dye in lymphocytes (MOLT-4) versus monocytes (U-937) /macrophages (U-937-DM). Positive hits were defined as fluorophores whose mean NIRF intensity for monocytes/macrophages were at or greater than five standard deviations above control lymphocytes. As a final hit, a cyanine-based Macrophage-specific near-infrared Fluorophore, MF800, was discovered with high specificity for monocytes/macrophages (Fig. S8 in File S1).

**Figure 1:**
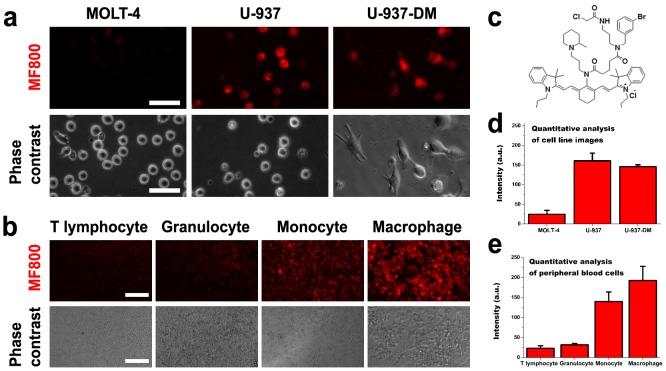
Selectivity of MF800 for monocytes/macrophages in a live-cell labeling assay. (**a**) Labeling of MF800 to MOLT-4 (lymphocyte) and U-937 (monocyte) cell lines, and in U-937-derived macrophages (U-937-DM) after incubation with 2 µM of the probe for 1 hour at 37°C. All NIRF images had identical exposure times and were normalized to the peak signal. Scale bars are 50 µM. (**b**) Probing human leukocytes collected from erythrocyte-lysed fresh blood and selected by magnetic separation. Images were obtained with the identical measurement and analysis conditions for NIRF after incubation with 6 µM of MF800 for 1 hour at 37°C. Scale bars are 100 µM. (**c**) Chemical structure of MF800. Image-based NIRF quantification from (**d**) the MF800 stained cell lines (n = 3, three different experiments) and (**e**) peripheral blood cells (n = 3, three different donor samples). Data are presented as mean ± s.d. a.u., arbitrary units.

MF800 shows an excellent fluorescence profile in U-937 monocytic cell lines and PMA-stimulated macrophages (Figs. 1a and 1d), as well as peripheral human blood monocytes and M-CSF-induced monocyte-derived macrophages (Figs. 1b and 1e). Figure 1c shows the chemical structure of MF800 and Figures S1–S5 in File S1 summarize the synthesis, characterization and physiochemical properties of MF800. MF800 (λ_ex_/λ_em_ = 806/825 nm) exhibits outstanding photostability compared to ICG, the only clinically-applicable NIR contrast agent, as previously reported [25]. The cytotoxicity of the MF800 was evaluated by a trypan blue dye exclusion assay in several cell lines until a 25 µM concentration (Fig. S6 in File S1), even though we could obtain the half maximum staining intensity even at 1.34 µM (Fig. S7 in File S1). No significant differences in cell viability were observed in the absence or presence of the MF800.

### MF800 enables *in vivo* detection of macrophage-rich paw inflammation

**Figure 2:**
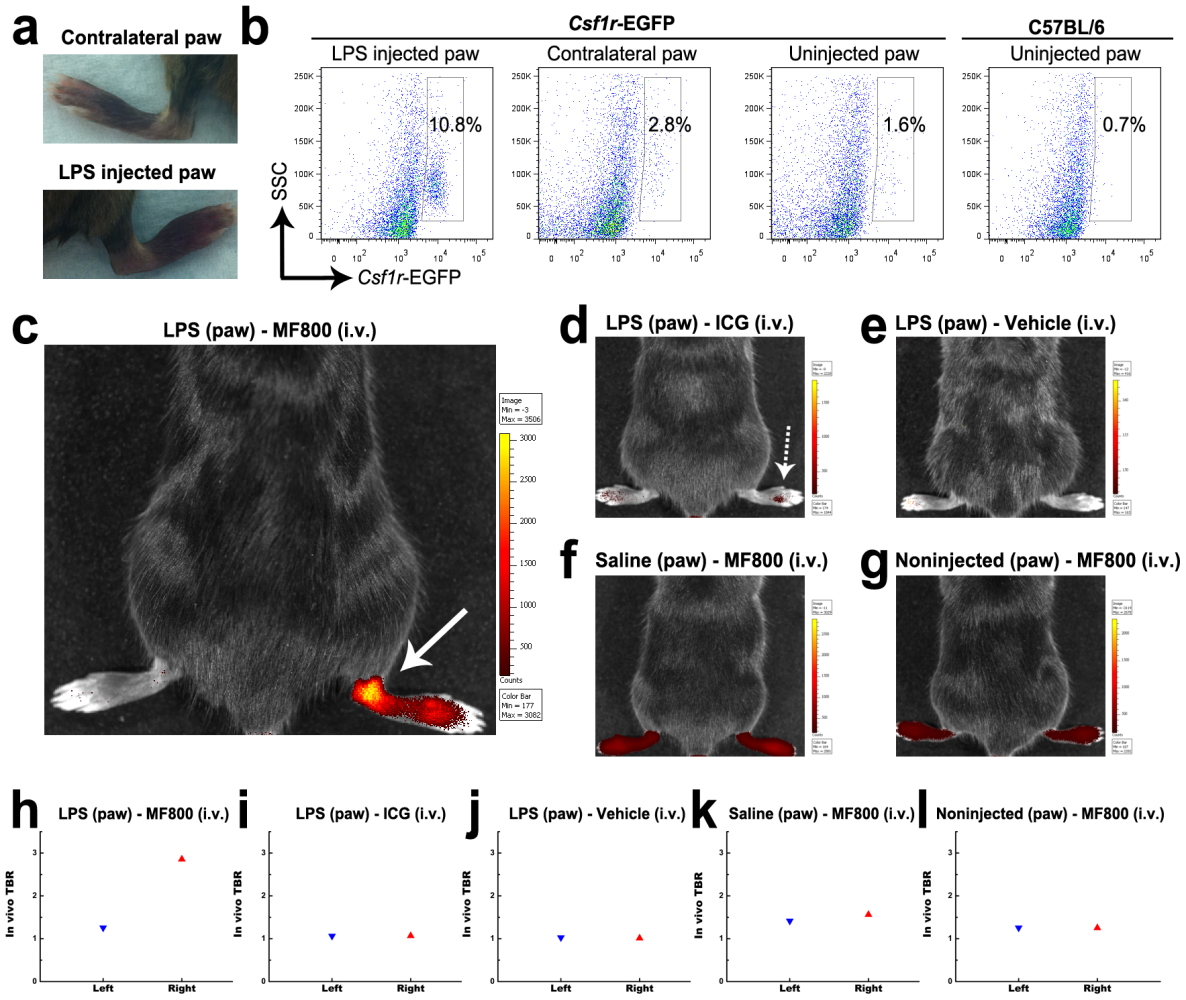
*In vivo* targeting of macrophage-rich inflamed paw with MF800. (**a**) Pictures of left control (top) and right LPS-injected (bottom) paws 24 hours after s.c. injection. Significant swelling of the ankle and the tarsus was observed in the right paw. (**b**) Flow cytometric dot plots of single-cell suspensions isolated from paw tissues of csf1r-EGFP mice in which macrophages express EGFP. Csf1r-EGFP-positive population constituted 10.8 % and 2.8 % of the total cells in the right and the left paws of LPS-injected transgenic mice, respectively, indicating recruitment of monocytes/macrophages to the inflamed right paw. Control paw-derived cells from non injected mice contained as 1.6 % and 0.7 % Csf1r-EGFP-positive cells from Csf1r-EGFP transgenic and naïve C57BL/6 mice, respectively. (**c–l**) *in* *vivo* NIRF imaging and signal quantifications of mice subcutaneously injected with LPS in the right paw, then i.v. injected with (**c, h**) MF800 (n = 6), (**d, i**) ICG (n = 2) or (**e, j**) vehicle control (n = 2). As controls, mice injected with (**f, k**) saline (n = 2) or (**g, l**) left uninjected (n = 2) were imaged after i.v. injection of MF800. Pictures shown are overlaid fluorescence and white light images, with corresponding color lookup tables 4 hours after i.v. injection. Note that MF800-mediated NIRF imaging highlights the macrophage-rich localized inflammation site (arrow) whereas ICG fails to resolve it (dotted arrow). The TBR value for the inflamed right paw in MF800-injected mice was higher than the contralateral paw, as well as ICG-injected and other control groups.

To explore whether MF800 could visualize macrophage-rich inflamed site *in* *vivo*, we utilized LPS-induced paw inflammation model. Twenty-four hours after s.c. injection of LPS in the right hind paw, mice received an i.v. injection of MF800 (n = 6), ICG (n = 2) or vehicle control (n = 2), and paws of live mice were imaged with an IVIS Spectrum imaging system. The mice saline-injected (n = 2) or non-injected (n = 2) in the right hind paw were also injected with MF800 and served as controls. The LPS-injected mice developed local swelling and redness in the right hind paw (Fig. 2a, bottom), but not in the left hind paw (Fig. 2a, top). A marked influx of macrophages in the right hind paw was seen by flow cytometric analysis of cells from the dissociated paw tissue of *Csf1r*-EGFP mice, in which EGFP is expressed in macrophages (EGFP positive cells: 10.8 % in the right vs. 2.8 %, Fig. 2b). As compared to ICG-injected mice that had very low NIRF signal (Figs. 2d and 2i), mice receiving MF800 showed robust NIRF signal in the inflamed right hind paw (TBR: 2.9 in the right vs. 1.3 in the left, Figs. 2c and 2h). All control animals exhibited experimental paw TBRs not significantly different than their contralateral paws, indicating that the signal from MF800 in the inflamed site was not likely an artifact, but due to the massive macrophage infiltration (Figs. 2e–g and 2j–l).

### Specific *in vivo* NIRF imaging of hind-limb ischemia with MF800

**Figure 3:**
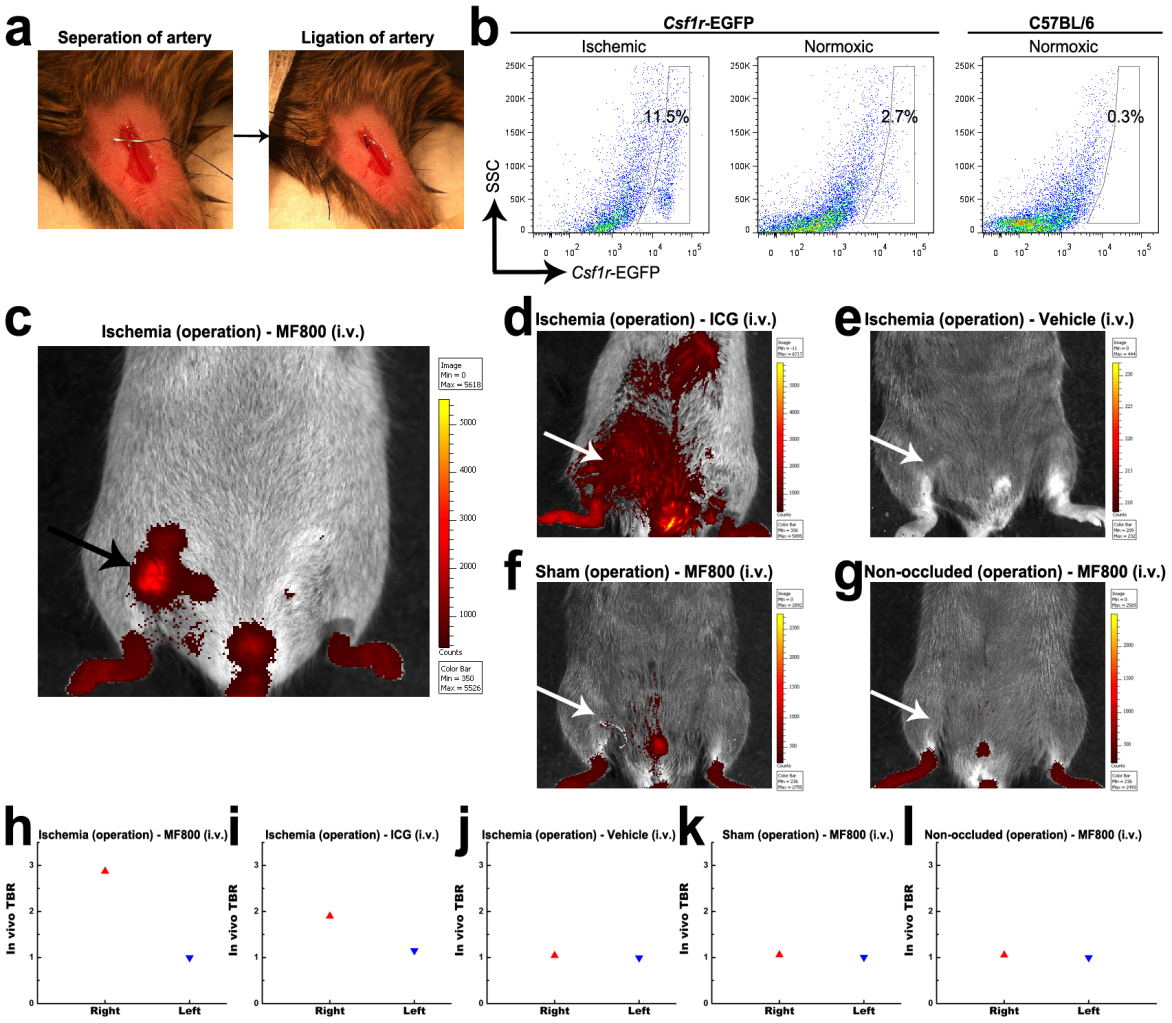
*In vivo* NIRF imaging of hind-limb ischemia with MF800. (**a**) To generate hind-limb ischemia, an incision was made in the skin, surgical thread was inserted underneath the femoral artery (left); then the right femoral artery was ligated by a triple surgical knot (right). (**b**) Flow cytometry analysis of digested tissues 3 days after ligation shows a higher proportion of csf1r-EGFP-positive macrophages in the ischemic right hind-limb (11.5 %, left) compared with the normoxic left hind-limb (2.7 %, middle) in csf1r-EGFP transgenic mice. Cells from the normoxic hind-limbs of naïve C57BL/6 mice were used for control (0.3 %, right). (**c–l**) *in* *vivo* NIRF imaging and signal quantifications of mouse models of hind-limb ischemia receiving (**c, h**) MF800 (n = 4), (**d, i**) ICG (n = 2) or (**e, j**) vehicle control (n = 2) intravenously. For negative controls, (**f, k**) sham-operated (n = 2) and (**g, l**) non-occluded (n = 2) groups were imaged after i.v. injection of MF800. Pictures shown are merges of pseudocolored fluorescence and white light images with corresponding color lookup tables 4 hours after i.v. injection. MF800 illuminated ischemic regions with significant macrophage recruitment (arrow) whereas ICGs were deposited nonspecifically. The TBR value for the ischemic right hind-limb in the MF800-injected mice was higher than that of the contralateral paw, as well as ICG-injected and other control groups.

We next investigated whether MF800 could identify not only simple inflammatory foci, but also ischemic lesions that contain pro-angiogenic macrophages *in* *vivo*. Mouse models of hind-limb ischemia were utilized by surgical ligation of the right femoral artery (Fig. 3a), which recruit angiogenic macrophages, to mimic critical limb ischemia in human patients. A significantly higher number of EGFP-expressing macrophages in the ischemic limb (11.5 %) compared with the normoxic limb (2.7 %) was confirmed by flow cytometry of enzymatically-digested tissues from the Csf1r-EGFP mice at day 3 after ligation (Fig. 3b).

*In vivo* NIRF reflectance imaging and signal quantifications were performed at 4 hours after i.v. injection of MF800 (n = 4), ICG (n = 2) or vehicle control (n = 2) using ischemic injury models (Figs. 3c–e and 3h–j). For controls, the sham-operated (n = 2) or normal (n = 2) mice were also imaged and analyzed after MF800 delivery (Figs. 3f, 3g, 3k and 3l). MF800-mediated fluorescence was localized to the macrophage-recruited ischemic region (TBR: 2.9 in the right vs. 1.0 in the left, Fig. 3c and 3h and Movie S1) whereas ICG-emitted fluorescence was distributed throughout the body, indicative of lower selectivity (TBR: 1.9 in the right vs. 1.1 in the left, Fig. 3d and 3i). Control animals showed minimal autofluorescence or artifacts (Figs. 3f, 3g, 3k and 3l).

### Intravital evaluation of MF800 macrophage targeting in *Csf1r*-EGFP mice

**Figure 4:**
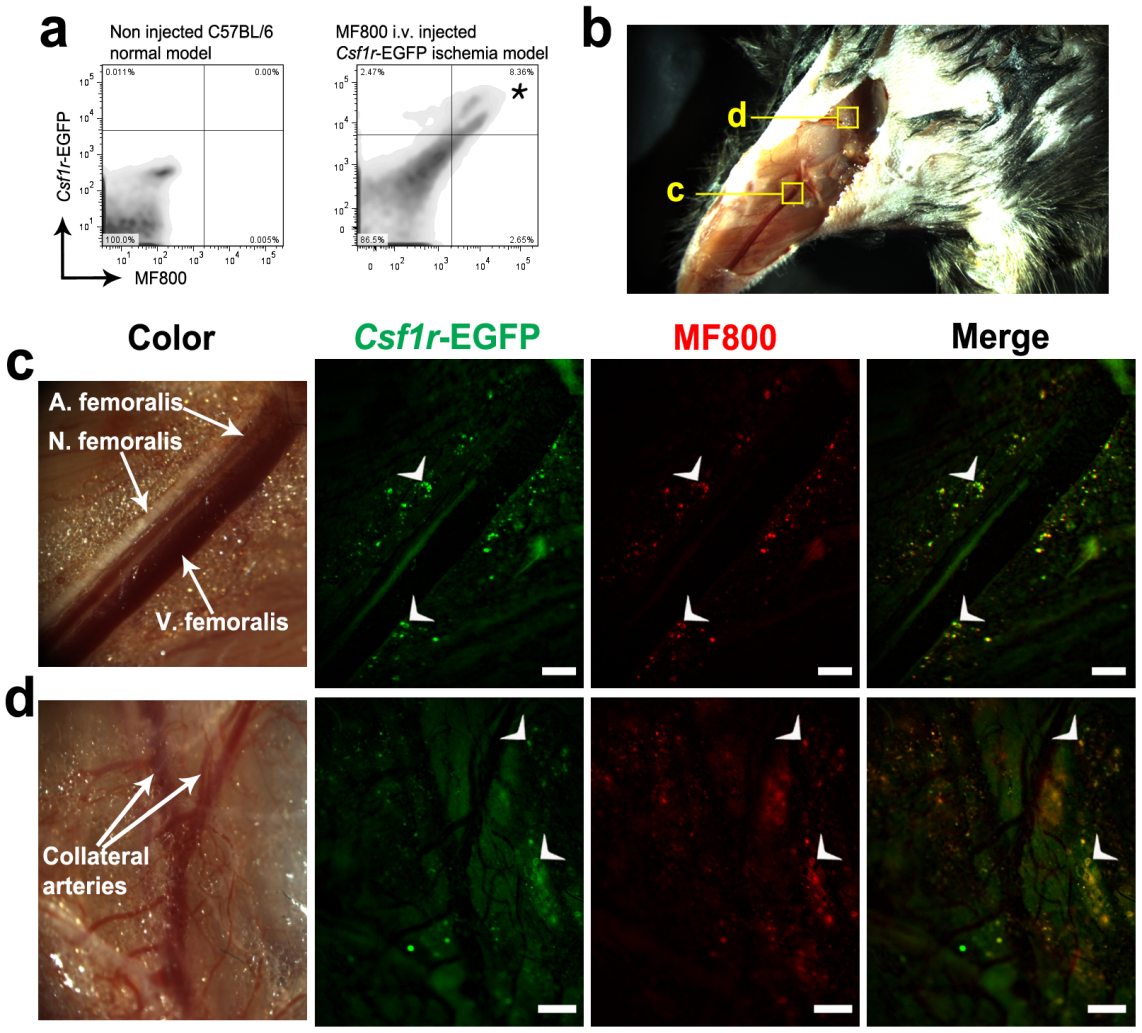
Intravital visualization of MF800 labeling to macrophages in csf1r-EGFP transgenic mice. (**a**) Flow cytometric plots of cells from the occluded artery tissues of csf1r-EGFP transgenic mice receiving an i.v. injection of MF800 3 days after ligation (right) and from healthy tissues of naïve C57BL/6 mice (left). The Csf1r-EGFP fluorescence (y axis) from macrophages correlated with MF800 intensity (x axis). Asterisk denotes co-localized cell population (8.36 % of total cells, 77.19% of EGFP^+^ cells). (**b**) A stereomicroscopic view of the ligated limb after removal of the skin and fascia, ventral aspect. (**c**) and (**d**) were taken from indicated regions. (**c**) Intravital NIRF imaging along the femoral artery displays MF800-labeled cells (pseudo-colored red), that co-localize with genetically-labeled macrophages (green) in csf1r-EGFP mice (merge). Scale bars are 200 µM. (**d**) A region of collateral arteries where arteriogenesis occurred shows prominent cellular infiltration of MF800^+^Csf1r^+^ cells, indicative of arterial growth and repair by macrophages. Scale bars are 1000 µM.

We next verified specific *in* *vivo* targeting of macrophages with MF800 at cellular resolution by intravital imaging of hind-limb-injured *Csf1r*-EGFP transgenic mice. In addition, flow cytometry of digested tissue from MF800-injected *Csf1r*-EGFP mice was conducted for quantitative analysis of macrophage-specific fluorescence at the single-cell level (Fig. 4a). EGFP-expressing macrophages highly correlated with MF800 positivity. An *in* *vivo* microscopic imaging analysis was also performed using a modified surgical NIRF imaging system to demonstrate cell-tracking capabilities. For the ischemic hind-limbs in *Csf1r*-EGFP mice, representative examples of intravital NIRF microscopy are depicted in Figures 4b–d. After removal of the skin and fascia (Fig. 4b), we were able to observe strong NIRF and EGFP signals adjacent to the femoral artery running along the ventromedial aspect of the leg, as well as in the vicinities of collateral arteries undergoing active arteriogenesis (Figs. 4c and 4d). As shown in the *in* *vivo* epi-fluorescence images in Figures 4c and 4d, MF800 NIRF co-localizes with macrophages expressing EGFP. Of note, non-specific background signals from autofluorescence were significantly suppressed in MF800-mediated NIRF compared to transgenic-labeling with EGFP. It has to be highlighted that there is no likelihood of crosstalk between EGFP and NIRF signals in both flow cytometry and intravital microscopy. There is no meaningful absorbance and emission of MF800 below 600 nm by light illumination at 480 nm, a peak excitation wavelength of EGFP (Figure S5b in File S1). Similarly, EGFP do not exhibit any absorbance and fluorescence in NIR region [29].

### Immunofluorescence characterization of MF800 labeled macrophages

**Figure 5:**
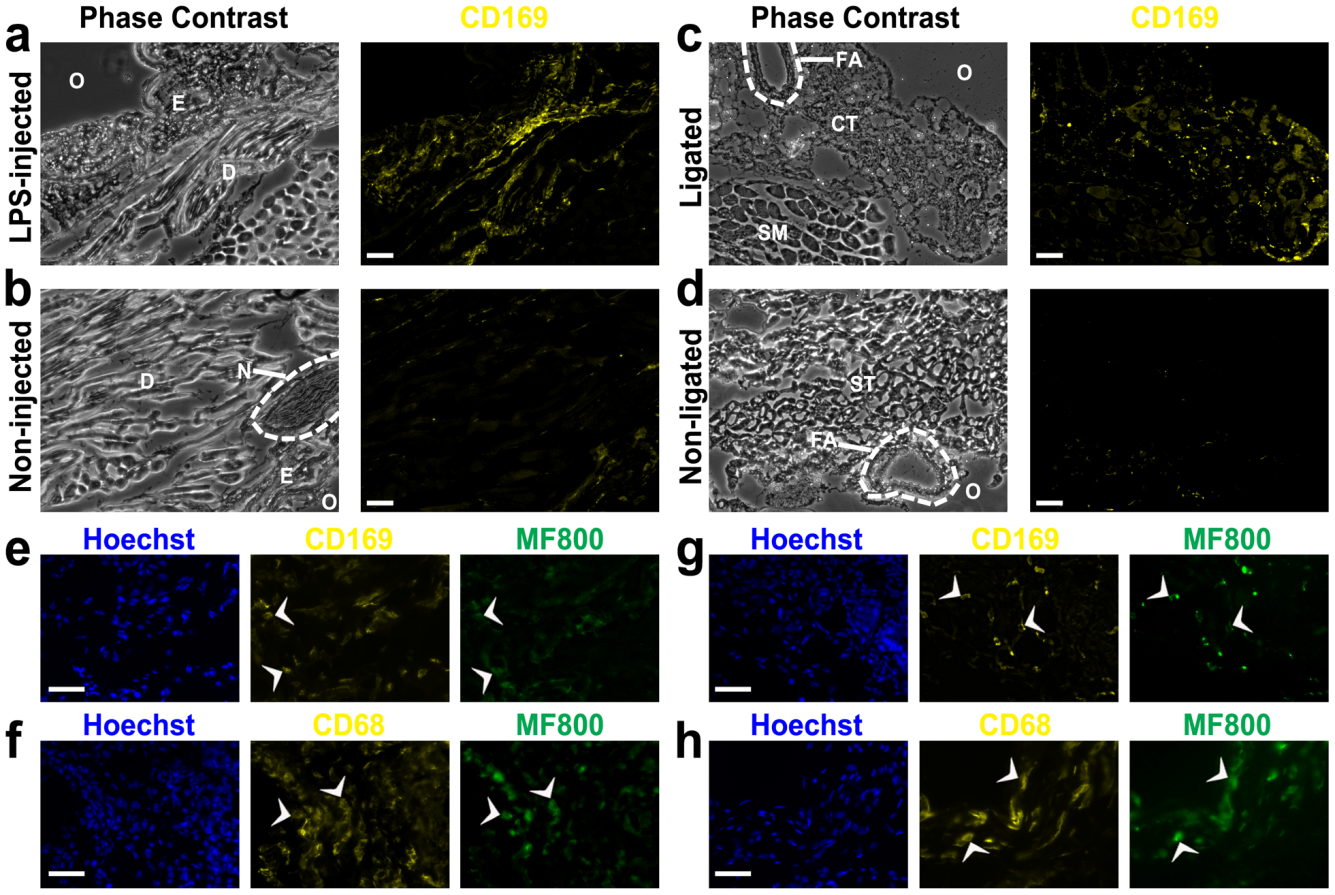
Immunofluorescence identification for *in vivo* labeling of macrophages with MF800 in an inflamed paw and an ischemic hind-limb. (**a-d**) CD169 macrophage-specific marker expression from regions that emitted (**a, c**) strong and (**b, d**) minimal MF800 signals *in* *vivo* from the paw inflammation (**a, b**) and the hind-limb ischemia (**c, d**) models. The strong *in* *vivo* NIRF regions were characterized by massive macrophage infiltration compared with the dark regions. Scale bars are 100 µM. O, outside of tissue; E, epidermis; D, dermis; N, nerve; FA, femoral artery; CT, connective tissue; SM, semimembranosus; ST, semitendinosus. (**e–h**) Characterization of cells labeled *in* *vivo* with MF800 using two macrophage-associated surface markers, CD169 and CD68 (yellow) from the LPS-injected paw (e, f) and the ligated paw (g, h). Co-expression of the selected markers on MF800^+^ cells (pseudo-colored green) was confirmed. Arrowheads indicate representative co-labeled cells. Scale bars are 40 µM.

Having determined the *in* *vivo* selectivity of MF800 for csf1r-EGFP-positive macrophages, we further characterized MF800-labeling cells with macrophage-specific markers using C57BL/6 mice. Real-time image-guided excision of fluorescent and non-fluorescent tissues was feasible in LPS-induced paw inflammation and hind-limb ischemia models using a NIRF surgical microscope. All excised tissue was analyzed for expression of the two macrophage-associated surface markers, CD169 and CD68. When compared at low magnification (x40, Figs. 5a–d), strongly fluorescing regions contained a high content of CD169^+^ macrophages whereas they were nearly absent in non-fluorescent tissues. As expected, most MF800^+^ cells co-expressed the markers CD169 or CD68 as shown in the several high magnification images (x400, Figs. 5e–h), further confirming the identity of these cells as inflammatory macrophages. Massive macrophage recruitment was seen mainly in the dermis for the paw inflammation model and in the connective tissue surrounding the femoral artery for the hind-limb ischemia model, in accordance with previous reports [10], [30].

## Discussion

Here, we present a new small-molecule NIRF probe capable of detecting macrophages *in* *vivo*. MF800 was identified from screening synthetic vital dye libraries and allowed us to track inflammatory macrophage infiltration in LPS-induced local inflammation. Furthermore, we showed that the use of MF800 offered specific and sensitive identification of *in* *vivo* ischemia through NIRF imaging and intravital microscopic visualization of angiogenic event. Direct comparison of MF800 with ICG, the only FDA-approved NIRF dye, indicated that MF800 has better *in* *vivo* selectivity for macrophages and superior imaging performance.

*In vivo* NIRF imaging is a new tool that bridges the gap between basic biological research and clinical practice. Due to recent significant advances in NIRF imaging technologies, it now offers broad potential applications to biomedical research as well as patient care [12], [13], [14]. One interesting application of NIRF is macrophage-specific imaging in many pathological conditions. Macrophages are the most plastic cells of the hematopoietic system and their pathophysiological roles in diseases have not been completely elucidated. Direct targeting of macrophages with *in* *vivo* non-invasive or minimally invasive NIRF imaging is unequivocally important to understand the great functional diversity of macrophages, and will ultimately lead to improvements in diagnostic accuracy and staging of macrophage-associated diseases.

In this study, we utilized a small-molecule-based strategy, focusing on preferential safety and toxicity profile, chemical diversity, conjugatability, and rapid *in* *vivo* targeting capability. Employing a high-throughput cell-based screening technique with photostable NIRF dye library developed previously [24], [25], we identified MF800, a macrophage-specific NIRF probe, and characterized its specificity through a cell-based assay, *in* *vivo* disease model imaging, intravital microscopy, and marker coexpression. In the current work, we focused on hind-limb ischemia with MF800 because imaging is relatively surface-oriented and macrophage density is indicative of disease status. Therefore, MF800-mediated NIRF imaging also has potential to be used for non-invasive macrophage tracking in monocyte/macrophage-based immunotherapeutic treatments in ischemia patients.

This new imaging technique has a few limitations for the detection of macrophages in ischemic tissues. First, the kinetics of MF800 in the ischemic regions and the optimal dose of MF800 to be injected will need to be evaluated in additional studies. Second, in comparison to other conventional radiological modalities, such as MRI and PET, NIRF imaging is currently limited to interventional/intraoperative use due to low tissue penetration. Future technological developments in optoacoustic detection or Cerenkov emission will help to circumvent these limitations [11], [16]. Lastly, rigorous evaluation for targeting range of MF800 in a whole hematopoietic system should be performed to maximize its potential benefit for various biological and clinical applications. The data presented above have shown specificity of MF800 for mononuclear phagocyte system against neutrophils (granulocytes) and lymphocytes in human blood and molecular characteristics of MF800 targeted cells, *csf1r* ^+^CD169^+^CD68^+^, in inflamed and ischemic regions of mice. Yet, whether labeling affinity of MF800 is different with varying degrees of differentiation, origin of tissue or activation state remains to be elucidated. Accurate definition of macrophage is still controversial due to its marked phenotypic heterogeneity [31], [32], [33]. Of note, an urgent need exists for identification of specific molecular target to resolve the range of target cells. Studies using affinity pull down assay are currently underway to find out the protein binders of MF800.

## Conclusions

In conclusion, we showed *in* *vivo* detection of macrophages in mice using NIRF imaging after intravenous injection of a macrophage-targeted fluorochrome. This approach may have broad applications in biological research and clinical practice. *In vivo* imaging of macrophage infiltration using MF800-enhanced NIRF imaging may be helpful for the diagnosis and prognosis of several pathological states, including localized inflammation and critical limb ischemia.

## Supporting Information

File S1 **Supplementary Figures S1–S8, Supplementary Methods, and Supplementary References.**
(DOCX)

Movie S1 **Three-dimensional surface imaging of hind-limb ischemia by co-registration of the functional MF800 NIRF and anatomical X-rays.**
(WMV)
