# Supplementary material for: In Vivo Detection of Macrophage Recruitment in Hind-Limb Ischemia Using a Targeted Near-Infrared Fluorophore
Source: PLoS One. 2014 Jul 29;9(7):e103721. doi: 10.1371/journal.pone.0103721 (PMC4114964; doi:10.1371/journal.pone.0103721)
Supplement: File S1 — Supplementary Figures S1–S8, Supplementary Methods, and Supplementary References. (DOCX) [file pone.0103721.s001.docx]

***In vivo* detection of macrophage recruitment in hind-limb ischemia using a targeted near-infrared fluorophore**

Jung Sun Yoo^1,2^, Raj Kumar Das^2^, Zhi Yen Jow^3^, Young-Tae Chang^2,3*^

^1^Smart Humanity Convergence Center, Department of Transdisciplinary Studies, Graduate School of Convergence Science and Technology, Seoul National University, Suwon, Korea

^2^Department of Chemistry, National University of Singapore, Singapore

^3^Laboratory of Bioimaging Probe Development, Singapore Bioimaging Consortium, Agency for Science, Technology and Research, Singapore

*Correspondence to Y-.T. Chang

Laboratory of Bioimaging Probe Development, Singapore Bioimaging Consortium, Agency of Science, Technology and Research, Singapore 138667, Department of Chemistry and NUS MedChem Program of Life Sciences Institute, National University of Singapore, 11 Biopolis Way, #01-02, Helios Building, Singapore 138667, Phone: +65-6516 6774, Fax: +65-6779 1691, Email: [chmcyt@nus.edu.sg](mailto:chmcyt@nus.edu.sg)

**LIST OF SUPPLEMENTARY ITEMS**

**Supplementary Figure 1:** Synthesis scheme for MF800

**Supplementary Figure 2:** Identification spectra of MF800 obtained by using ^1^H-NMR

**Supplementary Figure 3:** Identification spectra of MF800 obtained by ^13^C-NMR

**Supplementary Figure 4:** Identification LC-MS spectrum of MF800

**Supplementary Figure 5:** Physiochemical properties of MF800

**Supplementary Figure 6:** Effect of MF800 on cell viability

**Supplementary Figure 7:** Flow cytometry based MF800 titration for U-937 monocytic cell line staining

**Supplementary Figure 8:** Schematic work flow of high-throughput screening

**Supplementary Movie 1:** Three-dimensional surface imaging of hind-limb ischemia by co-registration of the functional MF800 NIRF and anatomical X-rays

**Supplementary Methods**

**Supplementary References**

**Supplementary Figure 1:** Synthesis scheme for MF800. (a) DCM/ACN (7:1), NaH-CO_3_, RT, 2 h.

**
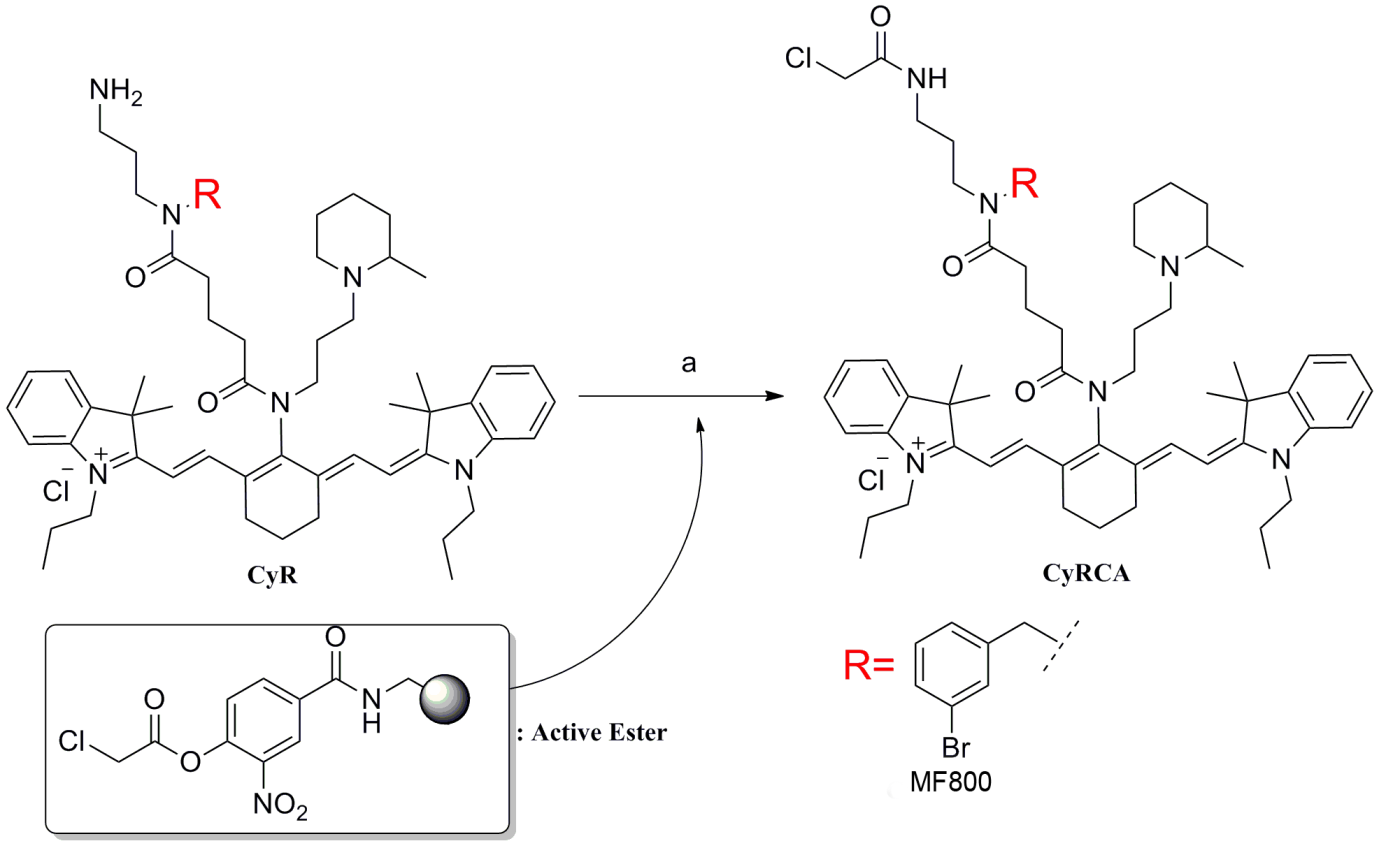
**

**Supplementary Figure 2:** Identification spectra of MF800 obtained by using 500 MHz ^1^H-NMR





**Supplementary Figure 3:** Identification spectra of MF800 obtained by ^13^C-NMR


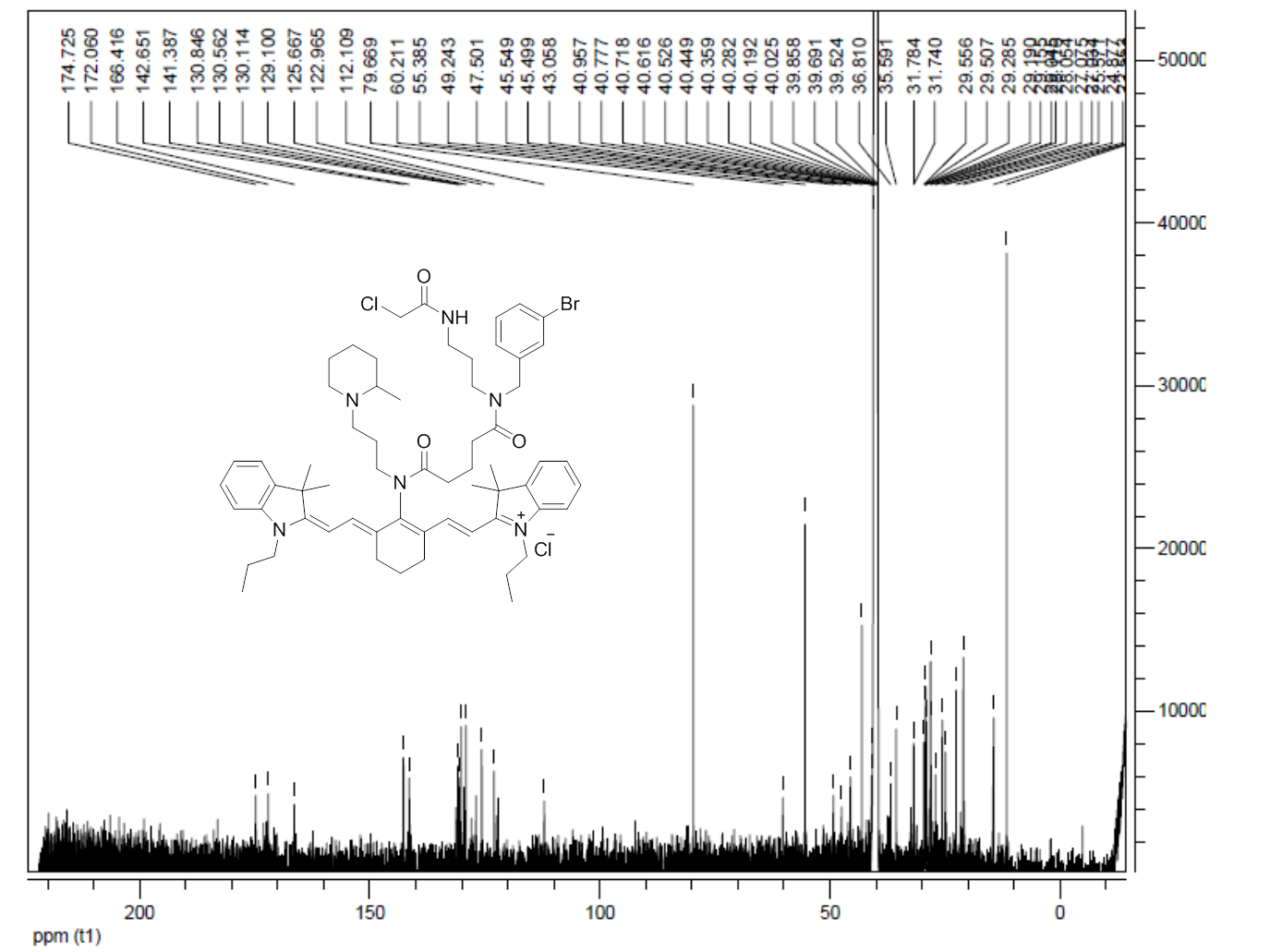


**Supplementary Figure 4:** Identification LC-MS spectrum of MF800

**Supplementary Figure 5:** Physiochemical properties of MF800. **(a)** Absorption and fluorescence emission spectra of MF800 with excitation at 740 nm. **(b)** Absorption and fluorescence emission spectra of MF800 with excitation at 480 nm. **(c)** Physicochemical properties of MF800.







**Supplementary Figure 6:** Effect of MF800 on cell viability. **(a)** MOLT-4 and HL-60 were treated with DMSO (control, 100%) at the indicated concentrations of MF800 for a 2-h, 4-h time period. **(b)** MOLT-4, HL-60, and U-937 were incubated with DMSO (control, 100%) or 1-μM MF800 for the indicated time periods. **(d)** MOLT-4, HL-60, and U-937 were incubated with DMSO (control, 100%) or 5-μM MF800 for the indicated time periods. Data represent mean viability values (%) + s.d. (n=3).










**Supplementary Figure 7:** Flow cytometry based MF800 titration for U-937 monocytic cell line staining. (a) U-937 monocytic cell lines were incubated with different concentrations of MF800 for 1 h and washed with PBS three times. The flow cytometry dot plot (left) and histogram (right) were acquired using a BD LSRFortessa. (b) The fluorescence intensity of the stained cells was analyzed from the flow cytometry results using BD FACSDiva software. Mean ± SD values of the average intensity were used to draw the graph, with the trend line added. The half-maximal staining intensity was obtained at 1.34 μM.

**
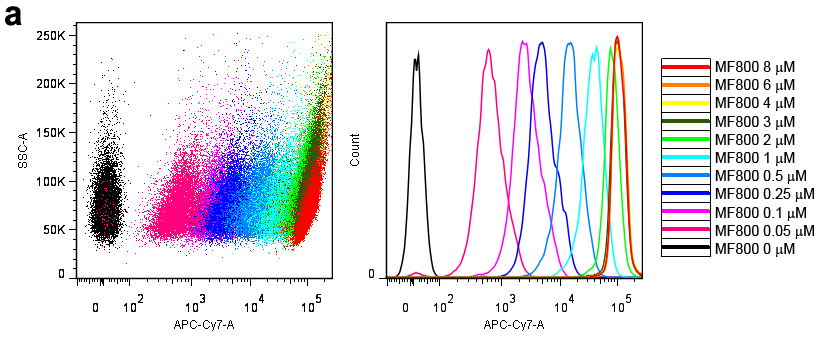
**

**

**

**Supplementary Figure 8:** Schematic work flow of high-throughput screening. (a) Cell-based high-throughput screening of NIRF small molecules (512 compounds) prepared by diversity-oriented synthesis. The 42 primary hit compounds were selected, using high-throughput flow cytometry of blood cell lines, based on the criteria that the mean values of the fluorescence intensities for monocytes/macrophages (U-937, U-937-DM) be greater than five standard deviations above the mean intensity of lymphocytes. The 10 secondary hit compounds were chosen by microscopic imaging of the same cell lines based on preferential staining ability and better imaging performance. Lastly, MF800 was selected as the final hit compound by the test with primary human blood cells. (b) Representative flow cytometry histogram of MOLT-4, U-937, and U-937-DMs after incubation with 1 μM of MF800. The mean intensity for each cell population was inserted.


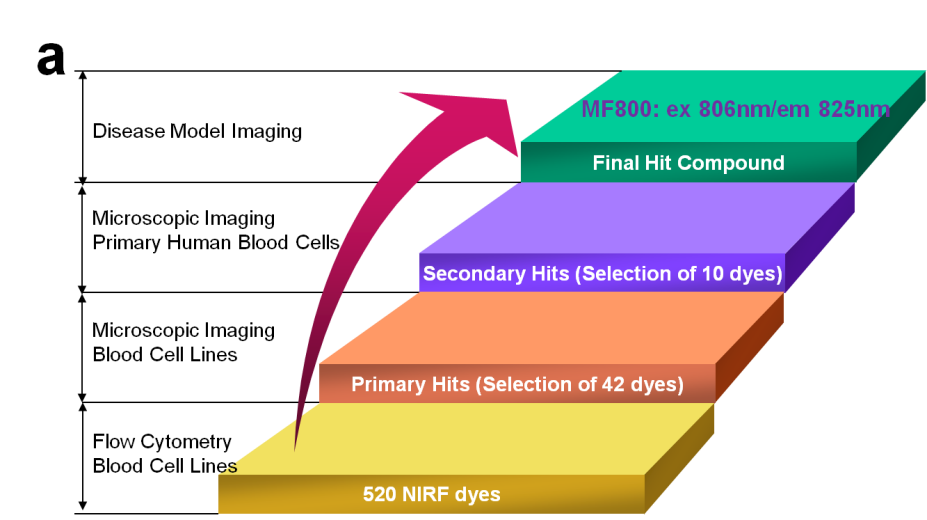


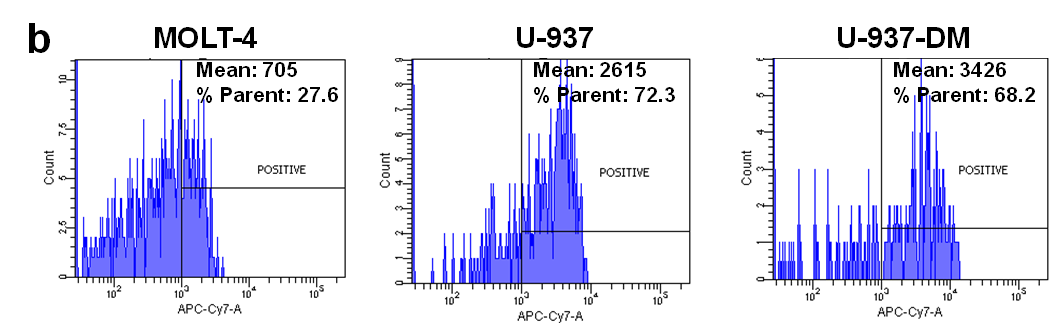


**Supplementary Methods**

**Reagents and materials for synthesis**

Amine building blocks and all other chemicals and solvents for the synthesis were purchased from Alfa Aesar, Fluka, Acros, MERCK, and Sigma Aldrich and were used without any purification. Merck Silica Gel 60 (particle size: 0.04-0.063 mm, 230-400 mesh) was used for the normal phase column chromatographic purification. From BeadTech Inc., Korea, 2-chlorotrityl alcohol resin (1.37mmol/g) was purchased. For analytical characterization of the compounds, HPLC-MS (Agilent-1200 series) with a DAD detector and a single quadrupole mass spectrometer (6130 series) with an ESI probe were routinely used. For the analytical processes, except as specified, the eluents were A: H_2_O (0.1% HCOOH); B: ACN (0.1% HCOOH), gradient from 0 to 100% B in 4 min; C_18_ (2) Luna column (4.6 x 50mm^2^, 5 μm particle size) used. ^1^H-NMR was recorded on a 500 MHz NMR spectrometer, chemical shifts are expressed in parts per million (ppm) and approximate coupling constants were calculated in Hz. Quantum yields and all other photophysical properties were measured using a SpectraMax M2 spectrophotometer (Molecular Devices) instrument and the obtained data were analyzed using Microsoft Office Excel 2007.

**Quantum yield measurements of MF800**

For the quantum yield calculation, we integrated the emission area of the fluorescent spectra and compared the value that for the same area of Cardiogreen as a standard (φ:0.13, in DMSO). The quantum yields are calculated using

Φ_x_ = Φ_st_(*I_x_/I_st_)(A_st_/A_x_*)(*η_x_^2^/η_st_^2^*) (1)

where Φ_st_ is the reported quantum yield of the standard, *I* is the integrated emission spectrum, *A* is the absorbance at the excitation wavelength, and *η* is the refractive indices of the solvents used. The subscript *x* denotes the unknown sample and *st* denotes the standard sample.

**Synthesis procedure and characterization of MF800**

To synthesize the MF800 compound, first, we synthesized the corresponding CyR compound according to the previously-reported procedure ^1^. Then, an active ester resin, about 200 mg, was suspended in 4 ml of DCM/ACN (7:1). Ten mg of MF800 compound was also dissolved in 1 ml of the same solvent, was added to the resin, and was shaken at room temperature. After 2 hour, the solution was filtered and dried in a fume hood. The crude compound was purified by column chromatography and 8 mg of product was obtained**:**

^1^H-NMR (500 MHz, CDCl3); δ1.05-1.58(m, 12H), 1.63 (s, 6H), 1.64 (s, 6H), 1.67-2.0 (m, 9H), 2.19-2.23 (m, 6H), 2.45-2.72(m, 6H), 2.91 (m, 2H), 3.16-3.36 (m, 11H), 3.68 (m, 2H), 3.98 (m, 4H), 4.14 (s, 2H), 6.10 (d, 1H, *J*= 14 Hz), 6.11 (d, 1H, *J*= 14 Hz), 7.06-7.39 (m, 11H), 7.53 (d, 1H, *J*= 11.5 Hz), 7.54 (d, 1H, *J*= 14 Hz), 7.71 (m, 1H).

^13^C-NMR (125 MHz, CDCl3): δ 174.72, 172.06, 166.41, 142.65, 141.43, 141.38, 131.28, 131.12, 130.84, 130.16, 129.48, 129.10, 127.12, 126.91, 125.66, 122.99, 122.96, 122.46, 122.06, 122.27, 112.10, 79.66, 60.25, 55.38, 49.23, 45.95, 45.92, 43.6, 37.66, 37.43, 37.22, 37.16, 36.92, 35.75, 32.13, 31.63, 31.67, 29.74, 29.70, 29.53, 29.44, 29.39, 29.35, 29.32, 29.32, 29.09, 29.04, 28.11, 28.20, 27.14, 27.00, 25.66, 24.99, 22.59, 21.05, 14.66, 14.60, 11.72

ESI-MS *m/z* (M ^+^), calc’d: 1073.5, found 1075.1 (due to chlorine and bromine isotope)

HRMS (ESI): calc for C_62_H_83_BrClN_6_O_3_^+^ 1073.5393; found: 1073.5438

**Synthesis of the active ester p-nitrophenol resin:** The nitrophenol resin was synthesized using a previously-reported procedure ^2^. This nitrophenol resin was subsequently treated with chloroacetyl chloride at room temperature with continuous shaking for two hours. The resin was then washed with DCM (X5) and dried under vacuum to obtain the active ester resin.

**Cytotoxicity assay of MF800**

The effect of MF800 on cell viability in MOLT-4, HL-60, and U-937 cells was determined using a trypan blue dye exclusion assay. Cell viability was expressed as the percent of each corresponding DMSO control (v/v). Briefly, 1×10^4^ cells in 1 ml of complete medium were plated in 12-well plates and allowed to grow overnight. The next day, cells were treated with the desired concentrations of MF800, and the plates were incubated for the desired time period at 37 ºC. At the end of the incubation, cells were collected and centrifuged at 5000 rpm for 5 min. The pellet was re-suspended in 30 μl of 0.4 % trypan blue solution in PBS, and live (white) and dead (blue) cells were counted using a hemacytometer under an inverted microscope.

**In vivo NIRF X-ray imaging of hind-limb ischemia**

Mice were anesthetized by intraperitoneal injection of ketamine (150 mg/kg) and xylazine (10 mg/kg). In the hind-limb ischemia models, the MF800s (100 μM in saline containing 1% of PEG and 0.1% of tween20, 10 μL/g) were injected intravenously (i.v.) 4 h before imaging experiments. The *in vivo* MF800-NIRF and corresponding X-ray anatomy images were taken on an In-Vivo MS FX PRO imaging system (Carestream, excitation: 745 ± 15 nm; emission: 800 ± 15 nm).

**Supplementary References**

1. Das RK, Samanta A, Ha HH, Chang YT. Solid phase synthesis of ultra-photostable cyanine nir dye library. *Rsc Advances*. 2011;1:573-575

2. Lee JW, Louie YQ, Walsh DP, Chang YT. Nitrophenol resins for facile amide and sulfonamide library synthesis. *Journal of Combinatorial Chemistry*. 2003;5:330-335
